# *Colletotrichum* Species Associated with Alfalfa Anthracnose: An Overview and Historical Perspective

**DOI:** 10.3390/microorganisms14010261

**Published:** 2026-01-22

**Authors:** Vojislav Trkulja, Tanja Vasić, Ranka Milašin, Nenad Trkulja, Slavica Matić, Milan Stević, Sanja Živković, Tatjana Popović Milovanović

**Affiliations:** 1 Agricultural Institute of Republic of Srpska, Knjaza Miloša 17, 78000 Banja Luka, Bosnia and Herzegovina; vtrkulja@blic.net; 2 Faculty of Agriculture, University of Banja Luka, Bulevar Vojvode Petra Bojovića 1A, 78000 Banja Luka, Bosnia and Herzegovina; rankamilasin@gmail.com; 3 Academy of Sciences and Arts of the Republic of Srpska, Bana Lazarevića 1, 78000 Banja Luka, Bosnia and Herzegovina; 4 Faculty of Agriculture, University of Niš, Kosančićeva 4, 37000 Kruševac, Serbia; vasic.tanja@ni.ac.rs (T.V.); zivkovic.sanja@ni.ac.rs (S.Ž.); 5 Institute for Plant Protection and Environment, Teodora Drajzera 9, 11040 Belgrade, Serbia; trkulja_nenad@yahoo.com; 6 Department of Agricultural, Food and Forest Sciences (SAAF), University of Palermo, Viale delle Scienze, 90128 Palermo, Italy; slavica.matic@unipa.it; 7 Faculty of Agriculture, University of Belgrade, Nemanjina 6, 11080 Belgrade, Serbia; stevicm@agrif.bg.ac.rs

**Keywords:** *Medicago sativa* L., fungal disease, *Colletotrichum* spp., epidemiology, control

## Abstract

Alfalfa anthracnose is an economically significant disease that leads to substantial biomass losses due to stem rot, reduced stand longevity, and a decline in forage nutritional quality. The disease is caused by multiple species within the genus *Colletotrichum*, including the 14 described species: *C. gloeosporioides*, *C. truncatum*, *C. lindemuthianum*, *C. destructivum*, *C. dematium*, *C. trifolii*, *C. medicaginis*, *C. graminicola*, *C. coccodes*, *C. sojae*, *C. spinaciae*, *C. lini*, *C. americae-borealis*, and *C. tofieldiae*. A thorough understanding of key aspects of the pathogen’s biology, along with its epidemiology, infection cycle, and accurate disease diagnosis, is essential for the development of sustainable management strategies. Knowledge of these factors allows us to anticipate disease outbreaks, implement timely interventions, and design integrated control measures that reduce reliance on chemical fungicides while maintaining crop productivity and forage quality. Although anthracnose management has traditionally relied on synthetic fungicides, this review synthesizes alternative control strategies to clarify the current state of knowledge and to provide new insights into the development of effective and sustainable approaches for managing *Colletotrichum* species.

## 1. Introduction

Alfalfa anthracnose (*Medicago sativa* L.) is a serious disease that can lead to significant biomass loss due to stem rot, reduced stand life, and impaired winter survival due to crown rot. Several *Colletotrichum* species are involved in the disease, negatively affecting biomass yield and the nutritional value of forage, thereby posing a significant threat to sustainable alfalfa production systems worldwide [1,2,3].

Understanding which specific species of the genus *Colletotrichum* infect alfalfa is crucial for developing effective disease management strategies [1,4]. Different species may vary in their virulence, host specificity, geographic distribution, and response to fungicides, which directly impact both yield and forage quality [2,3]. These differences directly influence the success of control strategies, the reliability of disease forecasting, and the development of resistant cultivars through breeding programs [2,3,4,5,6]. Accurate identification of these pathogens allows researchers and farmers to implement targeted control measures, predict disease outbreaks, and breed resistant cultivars [5,6]. Moreover, understanding the diversity of *Colletotrichum* species involved in alfalfa anthracnose is essential for assessing disease risks at regional and global scales and for mitigating the associated economic losses [5,7].

To support this effort, this review provides an overview and historical perspective on *Colletotrichum* species associated with alfalfa anthracnose worldwide, highlighting changes in their taxonomic concepts over time and summarizing current knowledge on their distribution, economic importance, symptomatology, life cycle, epidemiology, molecular identification, and management strategies [1,2,3,4,5,6,7].

## 2. Symptoms

**Figure 1:**
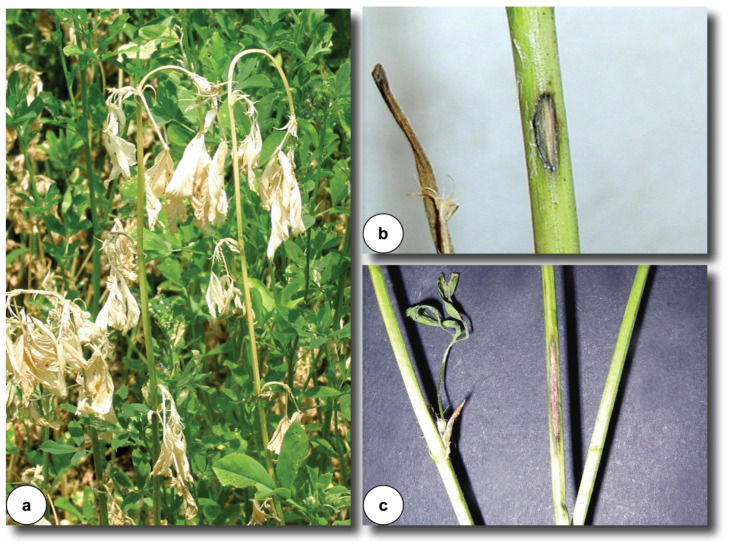
*Colletotrichum* spp. anthracnose symptoms on alfalfa are caused by (**a**) “shepherd’s crook,” (**b**) typical stem lesions, and (**c**) diamond-shaped stem lesions (natural infection) (photo by T. Vasić).

The most common symptoms of anthracnose in alfalfa plants are readily identifiable in the field by the appearance of straw-yellow to pearly discoloration on several stems, followed by wilting and the characteristic downward-curved stem tip, known as the “shepherd’s crook” (Figure 1a). Typical stem lesions are irregularly oval (Figure 1b) or diamond-shaped (Figure 1c), light to dark brown with dark edges, and often located on the lower third of the stem. Over time, disease progression leads to lesion enlargement accompanied by grayish-white in the center with dark brown to black margins. In later stages, lesions may coalesce and girdle the stem, causing wilting and eventual breakage at the site of infection. Small black fruiting bodies of the pathogen (*acervuli*) frequently appear within the lesion centers, producing numerous conidia under humid conditions [8,9,10,11].

In the initial phase of infection, small, watery black spots develop on the stem that gradually expand in a diamond-shaped or irregular pattern with a dark center. The upper portions of infected stems are often bent downward, leaves wilt, turn yellow and pinkish, and the entire stem dies, giving heavily infected fields a pinkish or straw-colored appearance. On the upper parts of the stems, smaller spots also appear under heavy infection, usually much smaller and often without sporulation. Lesion size and severity vary depending on host susceptibility, ranging from a few small, irregularly shaped blackened lesions on resistant stems to large, sunken, oval to diamond-shaped lesions on susceptible plants. These large lesions are straw-colored with distinct brown edges, and black *acervuli* (fruiting structures) appear in the bleached zones, detectable with a hand lens [10,11,12].

The fungal mycelium colonizes the stem downward, and crown anthracnose manifests once the affected stems dry out. Under severe infection, the whole branch may desiccate but remain erect. Infected crowns and roots show bluish-black or reddish-brown discoloration extending from the crown base into the taproot [13,14].

**Figure 2:**
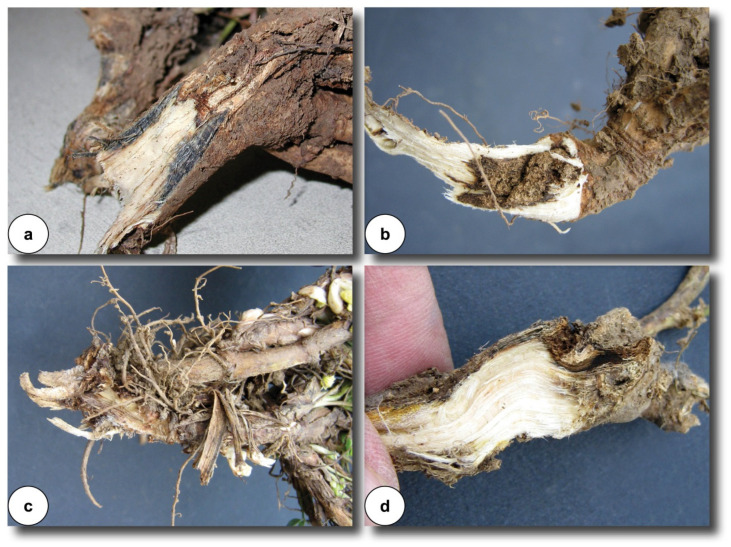
*Colletotrichum* spp. anthracnose symptoms on crown and root alfalfa caused by: (**a**) *Colletotrichum trifolii*; (**b**) *Fusarium oxysporum*; (**c**) *Phoma* sp.; (**d**) *Rhyzoctonia* sp. (natural infection) (photo by T. Vasić).

The most serious manifestation of anthracnose is the bluish-black crown rot phase, generally observed when killed stems are broken off at the crown (Figure 2a). In some cases, lesions are not visible on dead stems; however, a bluish-black discoloration at the stem base reliably indicates anthracnose. In contrast, if the base shows light brown discoloration, the disease may be *Fusarium* wilt (Figure 2b) or *Rhizoctonia* crown rot (Figure 2c), which can also occur simultaneously in the same field [12]. Likewise, a similar symptom is manifested in the case of *Phoma* crown rot (Figure 2d).

Between 2005 and 2010, extensive surveys of alfalfa fields in Serbia recorded a high incidence of anthracnose on stems and roots. The disease was most prevalent after the second cutting (July) in all six years of observation, regardless of weather conditions. It spread rapidly through neighboring plants, resulting in visibly yellowish-pearly patchy areas. Stunted regrowth following mowing was also characteristic of infection. The disease was typically most severe in stands in their second and third years of use, while in the year of sowing, symptoms were not observed under Serbian agroecological conditions [10].

As infection progresses, the pathogen invades the crown and root tissues. The mycelium grows downward through the stem, and after the stems dry, crown anthracnose develops. Crown and root infections are marked by dry rot and a bluish-black discoloration of infected tissues [10,14]. Crown infection may occur with or without stem lesions. Infected plants may die within one growing season or, if weakened, succumb to frost damage during winter [15,16,17,18]. In regions with warm and temperate climates, *Colletotrichum trifolii* and *C. destructivum* persist in the stems and crowns of alfalfa [17].

The formation of conidia and disease development are promoted by warm and humid weather, particularly during summer. The main propagules responsible for infection spread are conidia, which are dispersed primarily by rain splash. Dense stands, where plants are in close contact, exhibit higher disease intensity; tightly packed rows that touch each other reduce air circulation and increase moisture levels, creating favorable conditions for conidial dispersal. In such stands, the inoculum easily spreads from plant to plant, leading to widespread crown infection and the eventual death of plants [10].

Field surveys confirm that crown infection may occur even in the absence of stem lesions. In the central part of the root, just below the crown, light to dark brown diseased zones are visible and spread downward toward the root tip. Infected tissues frequently crack and die, resulting in plant wilting and death during the growing season or increased susceptibility to winterkill. By the fourth year of cultivation, stands severely infected with anthracnose become so thinned that further production for seed or silage is no longer economically viable [10].

Symptoms of anthracnose on alfalfa caused by different species of the genus *Colletotrichum* may vary. Among the 14 described *Colletotrichum* species, the characteristic shepherd’s crook symptom (Figure 1a) has been reported for *C. trifolii*, *C. destructivum*, *C. truncatum*, and *C. americae-borealis* [8,10,11,19], whereas this symptom has not been documented for the remaining species. Anthracnose symptoms caused by *Colletotrichum destructivum*, *C. americae-borealis*, and *C. linicola* also occur in leaves but are less common than in stems. Affected leaves develop small, irregularly shaped lesions that are slightly sunken, featuring a pale brown core with darker reddish-brown margins that expand to discolor larger tissue areas. Stem infection begins with small black lesions that enlarge and assume oval- to diamond-like shapes, leading to leaf abscission and stem wilting in severely affected plants [19,20,21]. In some cases, pale gray lesions with black borders and visible setae are observed on stems [21]. Typical stem symptoms caused by *C. truncatum* include large, sunken, irregular black lesions that appear about 12 days after inoculation and may girdle stems, resulting in wilting or breakage [22]. Similarly, according to Li et al. [23], in infections caused by *C. americae-borealis*, early-stage lesions are pale brown, prismatic or oval, with dark brown to black edges. As the disease progresses, lesions turn necrotic, their center become grayish-white with black specks, and in heavily infected plants, they girdle the stem, causing the above-ground parts to wilt or die.

The persistence of *C. trifolii* and *C. destructivum* conidia in crop residues and on the surface of harvesting equipment represents an important source of inoculum for subsequent sowings. The residual inoculum serves as a source of both secondary spread within infected fields and primary infection when favorable environmental conditions occur. In regions with cold winters, conidia of *C. trifolii* and *C. destructivum* can survive for up to 100 days [14]. Additionally, weed species that act as natural hosts for *C. destructivum*, as well as parasitic flowering plants such as dodder (*Cuscuta* spp.), contribute significantly to pathogen survival and dissemination [24,25].

Overall, anthracnose can appear at any time during the growing season and in stands of any age. Typically, it appears after the second cutting in stands two years or older, but it can also occur on seedlings before the initial cutting in early summer. The disease severely reduces plant vigor, causes stand thinning, and shortens the productive lifespan of alfalfa fields through the combined effects of stem lesions, crown rot, and winter mortality. The disease may result in plant death or reduced cold tolerance, with many plants failing to survive winter and resume growth in spring [8,10,11].

## 3. Geographical Distribution

In some areas, anthracnose poses a major threat to alfalfa (*Medicago sativa*), causing loss of total biomass due to stem death and reductions in stand longevity and winter survival as a result of crown rot. The disease is attributed to several *Colletotrichum* species.

**Figure 3:**
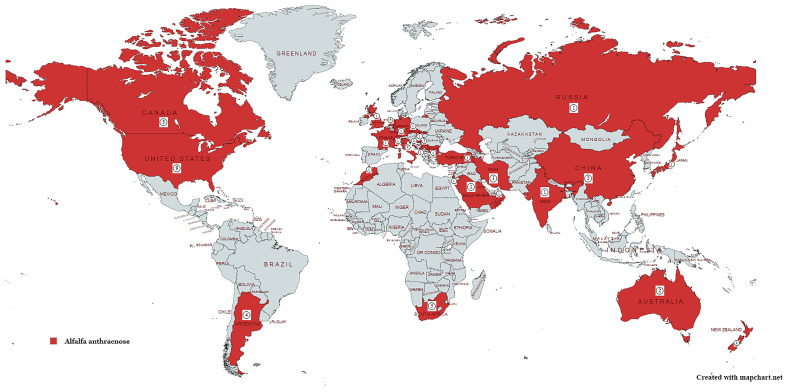
Global map showing the prevalence of alfalfa anthracnose (*Colletotrichum* spp.): 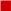 present; 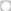 number in the circle present established *Colletotrichum* species per country (photo V. Trkulja).

Alfalfa anthracnose, caused by *Colletotrichum* species, has been reported across several countries in Europe [26]. The widespread presence of anthracnose pathogens has been documented in later studies from Italy [27,28,29], Croatia [30,31], the Czech Republic [28,32], France [28,32,33], Russia [28], the United Kingdom [34], Serbia [35,36,37,38,39,40,41,42,43,44,45,46], Montenegro [47], Turkey [22], the Netherlands [48,49], Bulgaria [32], Slovakia [32], Germany [6], and Switzerland [6] (Figure 3).

In Asia, species of the genus *Colletotrichum* that cause alfalfa anthracnose have been confirmed by research conducted in India [50], Oman [51], Saudi Arabia [29,52,53], Japan [54,55], Iran [56], China [11,20,23,57,58], and Israel [49] (Figure 3).

Alfalfa anthracnose caused by *Colletotrichum* spp. is also documented in North America: the USA [8,14,29,59,60,61,62,63,64,65,66,67,68,69,70,71,72,73,74] and Canada [75,76,77,78], and in South America in Argentina [19,21,65] (Figure 3).

In Africa, alfalfa anthracnose has been confirmed in South Africa [79,80,81] and Morocco [29,82] (Figure 3).

Anthracnose has also been confirmed in Australia [13,34] and New Zealand [51,53,83] (Figure 3).

The geographical distribution of *Colletotrichum* species reported to cause anthracnose of alfalfa, including the number of established species per country and literature references, is presented in Table 1.

Despite ongoing research, the full extent of alfalfa anthracnose caused by *Colletotrichum* spp. and its global distribution remains uncertain. Previous reports are particularly unreliable because they relied on morphology, pathogenicity assays, or the analysis of only a limited set of genes. Because these approaches are not fully dependable for accurate identification of *Colletotrichum* species, only findings yielded by molecular analyses (e.g., multigene phylogenetic analysis) should be relied upon when estimating the size and distribution of global *Colletotrichum* spp. populations [85].

## 4. Economic Impact

Globally, alfalfa ranks among the most important and widely cultivated perennial forage legumes, known for its exceptional biomass yield, high protein content, and broad ecological adaptability. Its global significance in agriculture derives from its significant biomass-yielding potential—exceeding 80 t ha^−1^ of green and nearly 20 t ha^−1^ of dry matter—and its high nutritional value, particularly crude protein and essential amino acids [86,87,88,89,90,91]. Alfalfa serves as a cornerstone in feeding programs for dairy and beef cattle, horses, sheep, and poultry [87]. It is cultivated across all 50 U.S. states and in numerous other countries due to its adaptability and superior forage value [92,93].

Among the many biotic constraints to alfalfa productivity, *Colletotrichum* anthracnose stands out among the most economically destructive diseases worldwide [21,68,94]. The disease affects both stems and crowns, resulting in substantial yield and quality reductions [62,68,95]. The first recorded observation of alfalfa anthracnose was made by Bain and Essary [59] in Tennessee, who reported widespread stand losses and described the pathogen, later identified as *C. trifolii*, as one of the most serious plant diseases in the state.

Over time, anthracnose has been documented in most areas where alfalfa is cultivated globally, encompassing the Americas, Europe, Africa, and Asia [10,21,57,76,81]. In the United States, the development of resistant cultivars by Devine et al. [96] significantly mitigated losses, with resistant lines yielding up to 10% more than susceptible parents [67,95]. However, the disease has re-emerged in new regions, particularly under humid or irrigated conditions, on older fields, and remains a major threat in North America, China, and South America [8,21,23,94].

Yield reductions attributed to anthracnose vary depending on cultivar susceptibility, pathogen species, and environmental conditions. In susceptible alfalfa genotypes, yield losses commonly range from 10 to 30% [9,17,21,46,97,98], though severe outbreaks can lead to stand mortality exceeding 80% [62]. In regions such as Nebraska, combined stresses from anthracnose, root rots, and adverse weather have caused complete stand collapse and losses exceeding 50% [8]. Anthracnose may occur at any stage of the growing season and affect stands of all ages [8]. In greenhouse conditions, alfalfa seedling mortality can reach up to 100% [11,99].

The anthracnose disease not only reduces biomass yield but also deteriorates forage nutritive quality. Infection leads to stem lesions, leaf loss, and reduced regrowth potential, resulting in decreases of 54.21–85.88% in the content of methionine, isoleucine, lysine, cysteine, and histidine levels. There was a pronounced negative correlation between crude fat, neutral detergent fiber, and the amino acids threonine, leucine, alanine, and tyrosine [57]. Infected alfalfa also shows a marked decline in forage nutritive quality, including a 35% reduction in crude protein content and lower in vitro digestibility [11,57,69]. Similarly, increases in cellulose content of up to 55% have been reported in Australia, negatively affecting hay digestibility [51]. Sherwood et al. [100] stated that infection of the leaves can have a negative effect on the health of farmed animals. The reduction in the assimilation surface, the loss of the leaves, the immaturity of the seeds, as well as the admixture of harmful metabolites in alfalfa, are the result of the presence of species of the genus *Colletotrichum* on the plants [10,13].

In China, anthracnose incidence in alfalfa fields has been reported in up to 75% of stands. Disease incidence varied between 7.5 and 53%, while mortality rates were up to 3%. Higher incidence was recorded in fields with older alfalfa stands [23]. Severe infections lead to patchy distribution and increased plant mortality. In Argentina, *C. americae-borealis* has emerged as a dominant causal agent of anthracnose, causing up to 25% yield loss during periods with extended rainfall or in irrigated areas and significantly affecting the forage industry [21,97,98]. In Serbia and other parts of southeastern Europe, *C. destructivum* and *C. trifolii* are responsible for widespread yield losses of 10–30%, depending on cultivar and climatic conditions [10,35,38,101]. The crown phase of anthracnose severely reduces stand longevity by killing crown buds and roots, decreasing the number of stems per plant, and reducing persistence [21,62,68]. Crown and root diseases caused by *Colletotrichum* spp. cause weakened plant vitality, increased sensitivity to frost, premature extinction, and increased weediness, all of which contribute to reduced hay quality [51]. In colder regions of northern Europe, however, the disease tends to be less economically significant [18].

The economic damage from *Colletotrichum* infections extends beyond direct yield losses. Reduced stand longevity necessitates early reseeding, increasing production costs. Lower-quality hay impacts livestock nutrition, leading to indirect losses in milk and meat production. Moreover, infected seeds reduce germination rates and limit international trade due to quarantine restrictions. Estimates in the USA suggest that one-quarter of the alfalfa hay crop and about 10% of the seed crop are reduced by diseases, with anthracnose being a major contributor [8]. The economic damage caused by the occurrence of anthracnose is difficult to express in monetary values because the plant is not a final product. When assessing damages from anthracnose infection, in addition to losses of green mass and alfalfa hay, losses related to the quality of meat and milk, as well as animal health, should also be calculated [10,51].

*Colletotrichum* anthracnose remains among the most impactful diseases of alfalfa from an economic perspective worldwide. It reduces forage yield by up to 30%, lowers nutritive value, and shortens stand life, with severe epidemics leading to total field loss. The disease’s economic significance is amplified by its indirect effects on livestock productivity, seed quality, and soil fertility contributions of alfalfa. Comprehensive management strategies combining resistant cultivars, crop rotation, and integrated disease surveillance are essential to mitigate these global losses.

## 5. *Colletotrichum* Species Causing Alfalfa Anthracnose

Available data show that alfalfa serves as a host for 14 *Colletotrichum* species, the agents responsible for anthracnose disease.

1. *Colletotrichum americae-borealis* Damm was initially isolated from alfalfa stems in Utah, USA, and described by Damm et al. [29]. This taxon belongs to the *C. destructivum* species complex [29]. To date, *C. americae-borealis* has been confirmed on alfalfa in the USA [29], China [23,57], and Argentina [21].
2. *Colletotrichum coccodes* (Wallr.) S. Hughes was first recognized as a pathogen in rotten potato tubers (*Solanum tuberosum*) in Germany and described as *Chaetomium coccodes* by Wallroth [102]. It was later renamed as *Colletotrichum coccodes* by Hughes [103]. To date, *C. coccodes* has been confirmed on alfalfa in South Africa [81] and New Zealand [51].
3. *Colletotrichum dematium* (Pers.) Grove was described by Persoon [104] as *Sphaeria dematium* from dead stems of *Urtica dioica* (stinging nettle), and it was later described as *Colletotrichum dematium* by Grove [105]. To date, *C. dematium* has been confirmed on alfalfa in Italy [27], the USA [61,70], the United Kingdom [34], and South Africa [81].
4. *Colletotrichum destructivum* O’Gara was first documented on clover (*Trifolium pratense*) and alsike clover (*T. hybridum*) stems and petioles in clover fields in the Salt Lake Valley, Utah, USA [106]. *C. destructivum* belongs to the *C. destructivum* species complex [29]. To date, *C. destructivum* has been confirmed on alfalfa in the USA [60,61,67], Croatia [31], South Africa [79,80,81], Serbia [29,36,46], Morocco [29,82], Canada [76,78], Saudi Arabia [24,29,53], Argentina [19], and Italy [29].
5. *Colletotrichum gloeosporioides* (Penz.) Penz. & Sacc [107] (teleomorph: *Glomerella cingulata* (Stoneman) Spauld. & H. Schrenk). *C. gloeosporioides* has a wide geographic distribution and infects a broad range of host plants [85,108]. To date, *C. gloeosporioides* has been confirmed on alfalfa in the USA [67,70].
6. *Colletotrichum graminicola* (Ces.) G.W. Wilson was first reported in 1852 as *Dicladium graminicolum* from infected corn and barnyard grass (*Echinochloa crus-galli* L.) plants [109]. Between 1852 and 1914, additional grass-infecting *Colletotrichum* species were proposed; however, Wilson later synonymized these taxa, including *C. cereale*, under *C. graminicola* [110] due to their similar morphology and shared grass hosts. To date, *C. graminicola* has been confirmed on alfalfa in the USA (as ‘*graminicolum*’) [60,67].
7. *Colletotrichum lindemuthianum* (Sacc. & Magnus) Briosi & Cavara is a species belonging to the *C. orbiculare* species complex [29]. It was described by Saccardo and Magnus as *Gloeosporium lindemuthianum* in *Phaseolus vulgaris* L. [111], and it was later renamed to *Colletotrichum lindemuthianum* by Briosi and Cavara [112]. To date, *C. lindemuthianum* has been confirmed on alfalfa in Canada [75] and Oman [51].
8. *Colletotrichum lini* (Westerd.) Tochinai was described by Tochinai [113]. The flax anthracnose pathogen was first described in the Netherlands by van Westerdijk [114] as *Gloeosporium lini*, referring to the genus as *Gloeosporium* (currently *Colletotrichum*). Tochinai [113] later transferred the species to *Colletotrichum* after studying multiple Japanese collections. No type specimen or precise collection site was reported by van Westerdijk. *C. lini* belongs to the *C. destructivum* species complex. According to Damm et al. [29], *C. linicola* is synonymous with this species. To date, *C. lini* has been confirmed on alfalfa in New Zealand (as ‘*linicola*’) [53,83], the USA [29], Serbia (as ‘*linicola*’) [44], and China (as ‘*linicola*’) [20].
9. *Colletotrichum medicaginis* Pavgi & U.P. Singh was first described by Pavgi and U.P. Singh [50] in stems of alfalfa. To date, *C. medicaginis* has been confirmed on alfalfa in India [50].
10. *Colletotrichum sojae* Damm & Alizadeh was isolated from the stem of *M. sativa* [74]. The species epithet is derived from the main host plant, *Glycine max*, soybean. It is a member of the *C. orchidearum* species complex [74]. To date, *C. sojae* has been confirmed on alfalfa in the USA, Iowa [74].
11. *Colletotrichum spinaciae* Ellis & Halst. was first described on living leaves of spinach (*Spinacia oleracea*) in the USA, New Jersey [115]. It belongs to the *C. dematium* species complex [55]. To date, *C. spinaciae* has been confirmed on alfalfa in the Netherlands [49]. Symptoms developed in hosts aside from spinach are generally weak [116].
12. *Colletotrichum tofieldiae* (Pat.) Damm, P.F. Cannon & Crous, was first identified as *Vermicularia tofieldiae* [117] from dead leaves of *Tofieldia* species in the eastern Tibet region, now part of southern Sichuan, China. Based on morphology, the fungus was initially assigned to a variant of *C. dematium* var. *minus* [118]. However, in 2009, Damm et al. provided strong evidence from ITS sequence analysis indicating that it belongs to the *C. destructivum* clade rather than *C. dematium* and subsequently renamed it *C. tofieldiae* [49]. Later, it was placed within the *C. spaethianum* species complex [55]. To date, *C. tofieldiae* has been confirmed on alfalfa in the Netherlands [49] and China [58].
13. *Colletotrichum trifolii* Bain was first described by Bain and Essary [59] in the USA as a species causing a novel clover anthracnose disease, which resulted in 25–75% yield losses in Tennessee and was also prevalent in Ohio and West Virginia. Described as *C. trifolii*, the pathogen affected stems, petioles, and rarely leaves of red clover (*Trifolium pratense*) and alfalfa (*M. sativa*), with additional occurrences documented in Kentucky and Arkansas [59,119]. Pathogen *C. trifolii* belongs to the *C. orbiculare* species complex [29]. To date, *C. trifolii* has been confirmed on alfalfa in USA [8,14,59,60,61,62,63,64,65,66,67,68,69,71,73], Croatia [30], Australia [13,55,65,120], Czech Republic, France, Italy, and Russia [28], Serbia [10,35,36,37,43,46,84], South Africa [81], Canada [77,78], Montenegro [47], Argentina [65], Japan [54,55], Netherlands [43,84], Switzerland [6], Germany [6] and China [11].
14. *Colletotrichum truncatum* (Schwein.) Andrus & W.D. Moore was first described as *Vermicularia truncata* by Schweinitz [121] in pods of *Phaseolus* in Pennsylvania, USA. It was later renamed *C. truncatum* [122]. It belongs to the *C. truncatum* species complex [55]. To date, *C. truncatum* has been confirmed on alfalfa in the USA [60,65,67], South Africa [81], Argentina [65], Australia [65,123], Turkey [22], the Netherlands [48], Israel [49], and China [11].

The 14 reported *Colletotrichum* species causing alfalfa anthracnose exhibit considerable variation in pathogenicity, ranging from highly aggressive, primary pathogens such as *C. trifolii*, *C. destructivum*, and *C. truncatum* to species that appear to act as weak or opportunistic pathogens with limited disease severity under field conditions [124,125,126,127]. Differences in host specificity are also evident, as several species (e.g., *C. gloeosporioides* sensu lato, *C. dematium*) exhibit broad host ranges, whereas others show a stronger association with leguminous or forage hosts [125,127]. In mixed infections, dominant species typically determine symptom expression, while secondary species may contribute to disease progression or persistence without being primary drivers of anthracnose development [11,126]. Notably, the pathogenic role of certain species, such as *C. spinaciae*, remains controversial due to inconsistent pathogenicity assays and limited host-specific evidence, highlighting the need for cautious interpretation and further experimental validation [11,125,127].

Beyond pathogenicity and host specificity, the global distribution of these *Colletotrichum* species reflects their ecological plasticity and adaptation to diverse climatic zones. Several taxa are prevalent in tropical and subtropical regions with warm temperatures and high humidity, which favor infection [125,126,127,128]. Conversely, species such as *C. truncatum*, *C. trifolii*, and *C. americae-borealis* demonstrate adaptability to a wide temperature range (4–35 °C), facilitating successful infections in temperate agroecosystems [11]. Geographic surveys further reveal distinct spatial patterns of species richness and host association, with pathogen assemblages varying by region and influenced by local environmental factors [124,128]. These observations collectively underscore that climatic conditions, particularly temperature and humidity regimes, are major determinants of species prevalence and adaptive mechanisms among *Colletotrichum* pathogens worldwide [11,124,125,126,127,128].

Species complex affiliation of the currently described *Colletotrichum* species causing anthracnose of alfalfa, as well as the relevance of their occurrence, is presented in Table 2.

## 6. Disease Cycle

Anthracnose of alfalfa (*M. sativa*) caused by species of the genus *Colletotrichum* is one of the most important fungal diseases of this forage crop. Anthracnose kills or severely injures both growing stems and new crown buds [17]. Characteristic symptoms appear as diamond-shaped lesions, straw-colored with brown margins, within which black *acervuli* form [9]. Under favorable conditions, these lesions enlarge, coalesce, and girdle the stem, leading to wilting and death of infected plants [46]. The disease is easily recognized in the field by the presence of a typical shepherd’s crook at the stem tip and diamond-shaped spots on the stem [11].

The pathogen profile of alfalfa anthracnose involves a complex of species with varying roles and virulence. While *C. trifolii* is the most aggressive primary pathogen, other species frequently co-occur. Isolates of *C. destructivum* and *C. dematium* f. *truncata* are pathogenic but often weakly virulent, appearing primarily as secondary invaders in stem lesions initially caused by *C. trifolii* [61,76]. Intriguingly, prior infection with these weaker pathogens can reduce the subsequent disease severity caused by *C. trifolii*, suggesting complex interspecific interactions. It is important to note the differences in lifestyle among *Colletotrichum* species: *C. trifolii*, one of the most common and aggressive alfalfa anthracnose pathogens and a member of the *C. orbiculare* species complex—whose representatives primarily infect plants in the Cucurbitaceae family, as well as numerous field crops and weeds—exhibits a predominantly biotrophic mode of development [29,129]. In contrast, many taxa within the *C. destructivum* species complex, including the main alfalfa pathogen *C. destructivum*, display pronounced internal differentiation, strong adaptation to leguminous hosts, and a hemibiotrophic lifestyle [119,130]. These differences in host specificity and complex membership have practical implications, as they can guide targeted disease monitoring, the selection of resistant cultivars, and the implementation of integrated management strategies for alfalfa anthracnose [131]. Virulence may also vary geographically; an isolate of *C. destructivum* from Ontario, Canada, was more virulent than mid-Atlantic U.S.A. isolates [61]. In some regions, such as southwestern Ontario, *C. destructivum* itself is considered the primary cause of anthracnose [76].

Historically, the disease has been more severe in the eastern than in the northern areas of America [12]. This geographical distribution has been partly attributed to the ability of the pathogen to overwinter. In northern areas, a reduced occurrence is due to the lack of overwintering capacity of *C. trifolii* effectively under harsh field conditions, though it can survive long periods in constant, cold storage. Ostazeski et al. [132] showed that *C. trifolii* survived in dry alfalfa stems for 10 months at −20 °C and was almost avirulent after four months when stored at 21 °C. The research of Lopez-Matos [133] and Barnes et al. [17] suggested that overwintering of *C. trifolii* occurs in the field in the stubble of infected alfalfa plants [14].

On alfalfa stems exposed to natural wetting and drying, *C. trifolii* survived for 100 days. In a dry, protected area at room temperature, the pathogen survived for at least 142 days. At constant temperatures of 25 °C, it persisted for 257 days. At 5 °C and at −15 °C, it was still present at low levels when the experiment was terminated after 853 days. The observation that pathogens can survive in infected stems for at least 100 days under field conditions highlights their role as an important source of secondary inoculum. However, when dispersed to new tissues during warm weather, its inability to survive longer than 100 days suggests that this material may not be significant as a source of primary inoculum. Apparently, the location of the lesion on the plant, i.e., in the crown, beneath the canopy, or in the canopy, does not affect the survival of the organism. The fungus did not survive for more than 100 days at either site. The data shows that the fungus survives at temperatures below freezing (−20 to −15 °C). The data could be interpreted as supporting a hypothesis that the fungus should survive as well in colder climates as in milder climates. However, this is not realistic, because in both cases the temperature and probably humidity were constant [14].

A critical means of long-distance dissemination and primary inoculum is via contaminated harvesting equipment. Debris on harvesting machinery can harbor the pathogen from one season to the next, serving as an important mechanism for spreading it from previously infected fields to new plantings [12]. The fungus may persist on the surfaces of mowing machines and in stacked hay, providing a vital pathway for the pathogen to spread from old, infected fields to new plantings [28,134]. In warmer regions, the fungus can survive perennially in the stems and crowns of living alfalfa plants, ready to initiate new infections when conditions become favorable [84].

With the return of warm, wet weather, the pathogen resumes active growth and sporulates. *Acervuli* with black bristle-like hairs (setae) develop in lesions on stems, and under favorable conditions these lesions enlarge, coalesce, girdle, and kill one or more stems [9,135]. On the plant surface, conidia of *Colletotrichum* spp. germinate and form specialized structures—*appressoria*—whose primary function is mechanical penetration through the cuticle and establishment of infection [136]. In rarer cases, degradation of the cuticle and penetration into internal plant tissues occur with the help of cuticle-degrading enzymes—cutinases [137]. In *C. trifolii*, melanization-related genes expressed in appressoria, particularly *PKS1*, are essential for the synthesis of dihydroxynaphthalene (DHN) melanin, which enables the generation of high turgor pressure required for direct mechanical penetration in alfalfa epidermal cells during the initial biotrophic phase. Conversely, pathogenicity of *C. destructivum* is less dependent on appressorial melanization and instead relies on the coordinated expression of secreted effector proteins, such as Cf-ECP1, which suppress host immune responses and facilitate intracellular colonization and maintenance of the biotrophic interface. This functional divergence illustrates distinct molecular strategies underlying interspecific pathogenic differentiation, with *C. trifolii* emphasizing penetration efficiency via melanized infection structures, whereas *C. destructivum* prioritizes effector-mediated host manipulation during infection of alfalfa [126,138,139].

After entry into the subcuticular space, most *Colletotrichum* species colonize epidermal cells via primary intracellular hyphae. At this stage, there are no visible symptoms on the host. Cell necrosis occurs with the formation of secondary necrotrophic hyphae. Pathogens that infect in this way are intracellular hemibiotrophs or facultative biotrophs [140]. From stem base lesions, the fungus penetrates crown tissues, with the crown rot stage marked by bluish-black discoloration of the invaded tissue [9,13,120].

Conidia are produced within *acervuli* on stem lesions and can be dispersed to petioles and stems by wind or splashing rain. Once infection occurs, hyphae grow within susceptible host tissues, leading to the formation of oval-shaped lesions. Under hot and dry conditions, infected stems may wilt and die. The fungus can extend down the stems into the crown and taproot, causing tissue death, increased susceptibility to winter damage, wilting, or plant mortality [10,141]. In warm and wet weather, spores form on stems and are spread by wind, splashing rain, or irrigation water, with some spores moving into the crown during wet conditions. The pathogen can also grow downward from stem infections into crown tissues. Movement of infected stems on machinery or wind-blown spores allows the disease to spread between fields. Notably, anthracnose is not a seed-borne disease [8].

A second mode of colonization occurs exclusively via subcuticular necrotrophic hyphae; in this mode, there is no transitional biotrophic phase, and necrosis of epidermal and mesophyll cells develops rapidly [137]. Latunde-Dada and Lucas [52] state that in the *C. destructivum*–host interaction, a hemibiotrophic phase is most often present, while Peres et al. [142] noted that, depending on the plant species, four basic infection modes are distinguished for *C. trifolii* and *C. destructivum*:

- Biotrophic development of the fungus, with formation of an *appressorium* on the plant surface and primary infection hyphae inside epidermal cells. Secondary conidia are produced directly from the *appressoria* and serve for further spread of the pathogen in nature. *C. trifolii* infects alfalfa plants in nature in this way [137].
- Subcuticular necrotrophic parasitism, with formation of an *appressorium* on the plant surface and development of secondary necrotrophic hyphae immediately beneath the cuticle. As the infection process advances, the secondary hyphae penetrate the intercellular space and cause necrosis. *C. trifolii* and *C. destructivum* cause infections in nature in this way [24].
- Hemibiotrophic development, with an *appressorium* on the surface and the formation of primary infection hyphae and later secondary necrotrophic hyphae inside epidermal cells. This infection type combines biotrophic and necrotrophic infection. *C. destructivum* infects by first, in the initial biotrophic phase, having intracellular primary hyphae delimiting individual epidermal cells, and in the subsequent necrotrophic phase, secondary hyphae attack neighboring cells—similarly to *C. higginsianum* (from *Brassicaceae* hosts) and *C. linicola* (from flax) [52,143].
- Combined hemibiotrophic and subcuticular necrotrophic parasitism, with an *appressorium* on the surface and formation of an infection vesicle inside plant tissue. *C. truncatum*, *C. linicola*, *C. destructivum*, and *C. higginsianum* infect host plants in nature in this way [52,143].

Spore adhesion and penetration begin when conidia become septate upon germination on the surface of alfalfa leaves and adhere to the host surface and germinate. Within 12 h, they form specialized melanized *appressoria*, whose primary function is the mechanical penetration of the host cuticle, enabling the fungus to establish initial biotrophic growth [24,136]. By 24 h after infection, epidermal cells of alfalfa leaves had been penetrated and contained intracellular fungal structures comprising swollen, saccate infection vesicles (or primary hyphae) with elongated neck regions. As infection progressed, the vesicles expanded through the formation of bulbous lateral protrusions and, by 48 h, developed into variably shaped, multilobed, multiseptate structures. In this phase of interaction, alfalfa leaves remained symptom-free, and multilobed vesicles were restricted to the initially infected epidermal cells, frequently densely occupying the cell lumen [24]. During this phase, which lasts approximately 48 h, the plant shows no visible symptoms. This represents a latent infection, a key survival strategy where the fungus and host coexist without visible changes. The ability to cause latent infection is a primary characteristic of species in the genus *Colletotrichum* [144]. During this metabolically reduced phase, the pathogen maintains basal metabolic activity and stress tolerance, while the expression of genes involved in host invasion and cell wall degradation is suppressed, thereby avoiding the activation of strong plant defense responses [145,146]. Reactivation of latent infections is often triggered by increased moisture and moderate temperatures, as well as by host physiological stress (e.g., senescence or mechanical damage), which promotes the transition to a necrotrophic phase and rapid symptom development under field conditions [147]. From an applied perspective, this dynamic indicates that alfalfa anthracnose management strategies should target not only visible symptoms but also the limitation of conditions conducive to latent infection reactivation, including optimization of agronomic practices, selection of tolerant genotypes, and precisely timed fungicidal or biological interventions [126].

The biotrophic phase dominated the first 48 h of the host–pathogen interaction and was quickly replaced by a necrotrophic stage, during which narrow secondary hyphae spread into adjacent leaf tissues. This phase was associated with the formation of water-soaked, expanding lesions and the development of sporulating, monosetate *acervuli* on infected plant surfaces [52]. After 48 h post-infection, bud-like outgrowths emerged at the margins of the multilobed vesicle and rapidly elongated to form invasive filamentous secondary hyphae.

Hyphae extended outward from the multilobed vesicles, penetrating the walls of infected cells and spreading into adjacent tissues, which resulted in the formation of water-soaked lesions on leaf surfaces. By 72 h post-infection, compact, knotted hyphal masses arose from the multilobed vesicles. These structures represented initials of *acervuli* and were present in abundance by 96 h after infection. Each mature *acervulus* contained a single melanized, septate seta surrounded by abundant conidia [24]. After 48 h, the fungus undergoes a dramatic shift to a destructive, necrotrophic mode. This transition is driven by environmental factors, increased temperature and air humidity, as well as chemical reactions in the host plant tissues [10,142,148,149]. Following 48 h of infection, bud-like outgrowths arose from the periphery of the multilobed vesicle and rapidly differentiated into invasive filamentous secondary hyphae. Cell necrosis occurs with the formation of secondary necrotrophic hyphae. These hyphae radiated from the multilobed vesicles, breached the walls of infected cells, and colonized surrounding tissues, forming water-soaked lesions on leaf surfaces. By 72 h post-infection, knotted hyphal aggregates developed from the multilobed vesicles, representing initials of *acervuli*, which became abundant by 96 h. Each *acervulus* contained a single septate, melanized seta surrounded by numerous conidia [24]. It is crucial to note that while *C. destructivum* is often a secondary invader, under warm, humid conditions, it can act as a sole pathogen, directly penetrating and infecting alfalfa leaves using this same hemibiotrophic process [19,24].

## 7. Epidemiology

Following the processes outlined in the disease cycle, the epidemiology of anthracnose on alfalfa caused primarily by *Colletotrichum* species is shaped by a complex interaction between environmental conditions, host physiology, and pathogen survival strategies. Understanding these relationships is essential for interpreting the temporal and spatial dynamics of disease occurrence in the field.

Infected stubble, stems, and crowns represent key reservoirs of inoculum that enable the fungus to persist between growing seasons, particularly in warmer regions [14,17,46]. As alfalfa is a perennial species, the pathogen can overwinter in these tissues and reinfect surrounding plants when temperature and humidity become favorable [46]. Although *C. trifolii* can survive on plant residues in milder climates, its ability to overwinter under field conditions is limited in colder regions such as the north-central United States of America and southern Canada [14,76]. Studies conducted in Serbia demonstrated that symptoms are rarely observed in the year of sowing but become increasingly frequent in the second and third years of production [10].

During warm and humid weather, the disease develops rapidly, reaching maximum severity between harvests in late summer and early autumn. The development and progression of alfalfa anthracnose caused by *Colletotrichum* species, particularly *C. trifolii* and *C. destructivum*, largely depend on infection temperature thresholds and the duration of plant tissue wetness, with moderate temperatures (≈20–28 °C) and prolonged periods of high relative humidity significantly increasing infection risk [99,142]. Shortening of the incubation period under favorable microclimatic conditions enables rapid symptom expression and a sharp increase in secondary inoculum, representing a critical window for timely intervention within integrated disease management frameworks [146]. Conidial dispersal is generally restricted to short- to medium-range distances within the crop canopy via rain splash and wind, resulting in spatially heterogeneous disease distribution and enabling targeted, localized control measures [150]. The disease reaches its highest severity during late harvests when warm daytime temperatures are followed by cool nights with heavy dew [8,12]. Epidemic development in the field is promoted by high temperatures (>27 °C) and relative humidity around 80% [10]. Under such conditions, anthracnose lesions appear on stems and leaflets, and sporulation is promoted by prolonged moisture from rainfall, irrigation, or morning dew [17,21]. The correlation between canopy density and infection intensity observed by Barnes et al. [17] further supports the role of prolonged leaf wetness in promoting disease spread. Dense stands retain surface moisture for longer periods, creating microenvironments conducive to sporulation and conidial dispersal.

The conidia of *Colletotrichum* species are disseminated primarily by splashing rain and wind [10,151], allowing them to readily adhere to the aerial parts of host plants [152,153,154]. Conidia are formed within *acervuli* surrounded by a water-soluble mucilage composed of high-molecular-weight glycoproteins, including some that are rich in proline. This mucilage contains compounds that inhibit germination as well as various enzymes, and it likely serves to protect conidia from desiccation and harmful plant metabolites [136,152]. Following dispersal, infection occurs when conidia germinate on moist tissues, typically at temperatures between 15 and 30 °C. Environmental factors play an important role in establishing infection and in anthracnose development in the host plant. The intensity and duration of rainfall, air temperature, infection potential, and pathogen dispersal significantly influence symptom expression [155].

Anthracnose incidence varies among growing regions. Severe outbreaks have been reported in the eastern and southeastern United States of America [17,62], while sporadic but sometimes damaging infections occur in northern regions during particularly warm and humid years [8,76]. Similar conditions have been observed in China, where disease prevalence and intensity correspond closely with periods of high temperature (26–32 °C) and moisture [11]. The distribution of *Colletotrichum* species infecting alfalfa is strongly shaped by climatic regimes and cropping patterns, with arid and semi-arid temperate regions favoring the dominance of well-adapted taxa, whereas humid environments tend to support higher species diversity [29,129]. For instance, the prevalence of *C. americae-borealis* in Argentina and Xinjiang, China, correlates with local arid to semi-arid climates and long-term, often continuous alfalfa cultivation, conditions that enhance pathogen persistence and regional specialization [29,57]. From an IPM perspective, incorporating climate- and cropping system–specific pathogen distributions into decision-support systems (DSS) can improve risk forecasting, optimize intervention timing, and support regionally tailored management strategies for alfalfa anthracnose [126].

Conidia surviving on dried plant debris or adhering to metal surfaces may initiate new infection centers following transport. This mechanism, along with splash dispersal, explains the frequent appearance of new infections distant from previously affected plants. Circumstantial evidence supporting this suggestion was provided by personal observation by Lukezic et al. [14] of the occurrence of anthracnose in an experimental field in northern Pennsylvania. That field had been harvested with an experimental harvester previously used on a field with infected plants located more than 100 miles away.

Although *C. destructivum* is generally regarded as a secondary pathogen [61,81], it can behave as a primary pathogen under warm and wet conditions in alfalfa, causing extensive foliar damage and reducing forage quality [19,24]. Species of *Colletotrichum* are capable of infecting both wounded and unwounded tissues and can persist epiphytically or endophytically in asymptomatic plants [148,149,156]. Moreover, the pathogen can survive on decomposing residues and alternative hosts, such as *Cuscuta* spp. and *Arabidopsis thaliana*, which may serve as inoculum sources during the growing season [10,24,157].

Temperature strongly influences the development and spread of the disease. The influence of temperature on the germination of *C. destructivum* spores was evaluated in sterile distilled water. After 48 h, spore germination was minimal at temperatures below 10 °C and above 30 °C. Within 24 h, 95–100% of spores germinated at temperatures between 15 and 25 °C, while germination at 30 °C nearly reached 100% after 48 h [60]. These temperatures overlap with those most favorable for alfalfa growth (20–25 °C), indicating that host and pathogen growth conditions coincide [11,158]. Findings by Zhou et al. [11] demonstrate that the temperatures favoring optimal pathogen growth and conidial germination closely match those optimal for host plant growth, meaning alfalfa plants are likely to be at risk of infection whenever *Colletotrichum* inoculum is present in the field during the growing season. As a result, infection can occur throughout the growing season wherever inoculum is present. This overlap also suggests that altering sowing or cutting dates could potentially reduce infection pressure, although further field validation is required.

The epidemiological impact of anthracnose extends beyond foliar and stem symptoms. Infected plants exhibit reduced vigor and yield and are often predisposed to winter injury. Field observations revealed that plants infected with *C. trifolii* exhibited 22–85% lower winter survival when assessed the following spring. Before dormancy, infected plants had 7% smaller roots and 29% fewer new crown buds, while levels of crown and root rot or root insect damage did not differ significantly [62]. These findings, along with reports of rapid disease spread and persistence in humid regions, confirm that anthracnose remains among the most important diseases affecting alfalfa production worldwide [11,17].

Overall, the epidemiology of *Colletotrichum* species on alfalfa reflects a balance between host susceptibility, environmental favorability, and inoculum availability. Understanding these relationships provides a foundation for developing integrated management strategies.

## 8. Molecular Characterization

As accurate pathogen identification is crucial for the development of an effective disease management strategy to overcome the challenges associated with identifying *Colletotrichum* spp., over the past few decades, traditional approaches have increasingly been integrated with molecular techniques. Modern studies commonly utilize polymerase chain reaction (PCR), RAPD fingerprinting, ITS region analysis of rDNA, and multilocus gene sequencing. In these analyses, species identification is typically confirmed through phylogenetic tree reconstruction based on sequences from multiple genes [85].

Historically, *Colletotrichum* classification relied on morphology and host range, but the scarcity of unique morphological traits has frequently caused misidentification due to limited morphological distinctiveness and unclear host specificity [55]. In recent years, multilocus sequence typing (MLST) has been widely used as the main approach for precise species identification [108]. Numerous studies have proposed comprehensive approaches to support the delimitation and classification of *Colletotrichum* species [55,159]. At present, multilocus sequence typing (MLST), targeting genes such as the internal transcribed spacer (*ITS*), actin (*ACT*), and glyceraldehyde-3-phosphate dehydrogenase (*GAPDH*), allows reliable classification and identification of both previously described and newly discovered *Colletotrichum* species [58,119,160].

It is recommended that multiple gene *loci* be characterized. To ensure accessibility, sequences should be deposited in recognized international databases [159]. Most frequently used genes for characterization of *Colletotrichum* species on alfalfa are internal transcribed spacer (*ITS*), actin (*ACT*), calmodulin (*CAL*), chitin synthase (*CHS-1*), *GAPDH*, glutamine synthetase (*GS*), histone H3 (*HIS3*), and β-Tubulin 2 (*TUB2*) (Table 3) [108,161,162,163,164,165,166,167,168,169,170].

Multilocus analyses have greatly improved the resolution of species identification within *Colletotrichum*. Prihastuti et al. [171] used six genes—*ACT*, *TUB2*, *CAL*, *GPDH*, *GS*, and rDNA-ITS—to study closely related *C. gloeosporioides* species (*C. gloeosporioides sensu lato*), showing clear separation of taxa. Multi-gene phylogenies have also clarified relationships among *Colletotrichum* spp. with curved conidia from graminicolous and herbaceous hosts [49,172]. Hyde et al. [127] compiled sequences from type and epitype cultures, providing a valuable resource for exploring species relationships [159].

According to Cai et al. [159], species delimitation requires the determination of the most suitable gene(s) for DNA barcoding, given that only one or a few genes can be selected once the taxa to be distinguished are known. A good approach would be to test and establish a selection of *loci* used by the different groups involved in *Colletotrichum* systematics to work towards the best barcoding gene. Unfortunately, different research groups have been utilizing different gene regions. For example, Crouch et al. [172] analyzed the ITS region together with *Apn2*, *Sod2*, and *Mat1-2*, while Prihastuti et al. [171] incorporated *ITS*, *CAL*, *GS*, *GPDH*, *ACT*, and *TUB2.* Johnston et al. [173] further expanded the marker set by including EF1α and CHS. For meaningful comparison across studies and to support future research on *Colletotrichum* diversity, it is essential to establish consensus regarding both the number and identity of gene regions to be sequenced. Equally critical is the consistent interpretation of phylogenetic groupings within a taxonomic framework. At present, a universally accepted species concept is lacking; however, the concept of “genealogical concordance” has increasingly been adopted to delimit phylogenetic species [174]. Accordingly, Cai et al. [159] proposed that species status should be assigned to well-supported, resolved phylogenetic lineages that are consistent with distinguishable phenotypic traits.

Within the *Colletotrichum* species, *C. americae-borealis* and *C. lini* share identical ITS and *GAPDH* sequences; however, they can be reliably differentiated from other species in this complex using *ACT*, *HIS3*, *CHS-1*, and *TUB2* loci. *Colletotrichum lini* is likewise distinguishable based on variation in *ACT*, *CHS-1*, *HIS3*, and *TUB2.* According to Damm et al. [29], *C. linicola* was synonymized with this species. In contrast, *C. destructivum* can be separated from related taxa using *ACT*, *ITS*, *HIS3*, and *TUB2* sequences, although its *GAPDH* region is identical to that of *C. ocimi* [29]. Similarly, *C. sojae* can be identified by differences in *ACT*, *HIS3*, and *TUB2*, whereas its *ITS* sequence is indistinguishable from that of *C. vittalense* [74]. For C. *coccodes* and C. *graminicola*, *ITS* regions were successfully amplified using the ITS1 and ITS4 primer pair [175].

Although *C. trifolii* and *C. malvarum* share identical *ITS* and *CHS-1* sequences, they can be differentiated based on other genetic loci (*GAPDH*, *HIS3*, *ACT*, *TUB2*, *GS*), with GS sequences being the most informative [119]. The *Colletotrichum orbiculare* species complex comprises *C. trifolii* and seven closely related species, all of which are plant pathogens with specific herbaceous host ranges. Species within this complex can be reliably identified using *GS* sequences alone [119]. The *Colletotrichum destructivum* species complex includes *C. destructivum* and 14 related species, primarily plant pathogens, which can be distinguished using a combination of *TUB2* and *GAPDH* loci. Within this complex, *C. destructivum* is further separable from related taxa by *ACT*, *HIS3*, *ITS*, and *TUB2* sequences, although its *GAPDH* sequence is identical to that of *C. ocimi* [29]. The *Colletotrichum gloeosporioides* species complex consists of *C. gloeosporioides* and 37 closely related plant pathogenic species. ITS separates *C. gloeosporioides* from all other species [108]. The *Colletotrichum dematium* species complex comprises *C. dematium* and 10 closely related species, which are primarily associated with temperate climates [55]. Species within this complex can be differentiated using *ACT*, *CHS-1*, *GAPDH*, *ITS*, and *TUB2* sequences [73]. Non-pathogenic isolates of *Colletotrichum* spp. may generally be distinguished from virulent plant pathogens through a combination of molecular markers, host inoculation assays, and assessment of symptom development under controlled conditions [118].

An overview of species-core identification genes, PCR primers, and literature references used for molecular identification of *Colletotrichum* species reported to cause anthracnose of alfalfa is presented in Table 4.

## 9. Control

The control of *Colletotrichum* anthracnose in alfalfa, as in other plant species, is based on integrated management combining cultural/ecological practices, host resistance, biological control agents, physical/diagnostic measures, and judicious fungicide use [179,180,181,182,183]. In most production systems, resistant cultivars are regarded as the primary control measure, with fungicides and other tactics used to protect stands where resistance is incomplete or pathogen populations are changing [1].

### 9.1. Cultural and Ecological Management Practices

#### 9.1.1. Resistant Cultivars and Breeding for Resistance

For disease management, cultivating alfalfa varieties resistant to anthracnose is widely recognized as the most effective strategy to reduce forage and stand losses. Growers are strongly advised to use numerous varieties that are resistant or highly resistant to anthracnose, as well as to other key diseases such as *Phytophthora* root rot [8].

Early recurrent phenotypic selection work by Devine et al. [96] showed that three cycles of selection in the greenhouse and laboratory could markedly increase the frequency of highly resistant plants, raising it from 5%, 5%, and 1% to 82%, 72%, and 59% in the cultivars Glacier, Saranac, and Vernal, respectively, and from 20% to 88% in the experimental population MSHp6F. The highly resistant class of plants was defined by a lack of lesions and *acervuli*, and the authors concluded that recurrent phenotypic selection in controlled conditions was very effective in raising anthracnose resistance levels in alfalfa populations [96].

Later work in Australia by Irwin et al. [1] confirmed that breeding lucerne for resistance to diseases and pests is an effective strategy to reduce productivity losses. Among the diseases affecting lucerne, anthracnose, caused by *C. trifolii*, is one of the most significant, impacting both stand persistence and forage yield. The primary method of controlling anthracnose is the cultivation of resistant cultivars, and selection for stem anthracnose resistance has also been shown to provide protection against crown rot. Genetic analyses have identified both dominant and quantitatively inherited resistance in lucerne: in North American germplasm, the resistance to race 1 of *C. trifolii* is conferred by the dominant gene *An1*, while *An2* confers resistance to both races 1 and 2, whereas Australian cultivars such as Trifecta and Sequel carry more complex forms of resistance that do not fit simple tetrasomic models [1,4,184].

*Colletotrichum trifolii* and *C. destructivum* employ distinct pathogenic strategies, resulting in differential activation of host resistance mechanisms that may be either qualitative (race-specific) or quantitative (partial, polygenic). Accordingly, screening of alfalfa resistance sources should combine species-specific inoculation assays with the evaluation of multiple disease components, allowing discrimination between strong, isolate-specific resistance effective against *C. trifolii* and broader, quantitatively inherited resistance more relevant for taxa within the *C. destructivum* complex [99,131]. The integration of molecular markers associated with hemibiotrophic phase transitions and host recognition further enhances the identification of durable resistance sources [126]. Such criteria enable targeted deployment of resistant cultivars and more precise integration of host resistance into anthracnose control strategies.

In the sequel clone W116, resistance to *C. trifolii* race 1 is incompletely recessive and quantitatively inherited, and QTL mapping identified a multi-locus region on linkage group 4 that plays a significant role in conferring the resistance phenotype. DNA markers linked to these QTL are proposed as tools to identify and deploy this quantitatively inherited resistance in lucerne breeding programs, particularly because such incompletely recessive resistance is difficult to exploit efficiently using phenotypic selection alone [1].

Field and greenhouse work illustrate how resistance varies among commercial cultivars. In long-term greenhouse inoculation tests, red clover varieties showed higher average survival to *C. trifolii* than alfalfa varieties, and field data for alfalfa were too limited to assign official susceptibility rankings; annual *Medicago* species were highlighted as potential gene sources to improve *M. sativa* for anthracnose resistance [5,6].

Serbian studies showed that cultivars Florida 77, Vernal S, and Banja Luka were resistant to the tested *C. trifolii* isolates, while K-1, K-28, Osječka 12, NS Slavija, and Zaječarska 83 were highly susceptible. Banja Luka also showed resistance to tested *C. destructivum* isolates, whereas K-28, K-1, NS Slavija, and Zaječarska 83 were highly susceptible; several American cultivars (Affinity 401 + Z, Vernal S, Florida 77, Perry) also showed significant susceptibility to *C. destructivum*. Overall, these studies demonstrate substantial genetic variation in anthracnose reaction and support the use of resistant or less susceptible cultivars as a central cultural control measure. Genotypes with the lowest susceptibility identified in screening programs are intended for use in the selection and improvement of alfalfa for resistance [10,45].

#### 9.1.2. Crop Rotation and Sanitation

Extension recommendations emphasize that fields previously affected by alfalfa crown rot and anthracnose should be planted with non-host crops for a minimum of two years before re-seeding alfalfa to reduce survival of inoculum in crowns and debris. Because the anthracnose fungus persists in infected plant debris, stacked hay, and on harvesting equipment, removing plant debris from harvesting equipment prior to the first spring cutting and periodically throughout the season and mowing young stands before older stands is recommended to help prevent the introduction and spread of the pathogen into clean fields. Avoidance of stress (e.g., over-cutting, nutrient stress, and waterlogging) is also advised as part of good crop management to reduce disease losses, since anthracnose interacts with other diseases and environmental factors to produce cumulative stress leading to yield and stand decline [8].

#### 9.1.3. Other Ecological Approaches

A review of lucerne diseases notes that disease management currently uses “eco-friendly and chemical approaches,” but that chemical measures are mostly applied and can induce resistance in pathogens and cause environmental hazards, reinforcing the need to integrate more ecological and non-chemical tactics into control programs [7].

For anthracnose specifically, based on the available lucerne literature, integrated management principles from other *Colletotrichum* pathosystems (e.g., attention to microclimate, canopy density, and moisture period) can be extrapolated, but direct data for practices such as biofumigation, anaerobic soil disinfestation (ASD), intercropping, or soilless culture in alfalfa are currently limited; these remain areas for future experimental work.

### 9.2. Biological Control Strategies

Biological control is increasingly recognized as a promising component of anthracnose management in alfalfa. Hu et al. [3] state that biological control represents a promising, environmentally friendly strategy for disease management. In the same study, *Bacillus amyloliquefaciens* indigenous strain LYZ69 exhibited potent in vitro inhibition of *C. truncatum*, with a 20% (*v*/*v*) cell-free culture causing 60% mycelial growth reduction and complete inhibition of spore germination. Under greenhouse conditions, the biocontrol strain LYZ69 conferred 82.59% protection to alfalfa, only slightly lower (by 12.92%) than that observed for the chemical fungicide chlorothalonil in identical conditions. The application of strain LYZ69 reduced disease incidence from 100% in the control to 55.32% in the treatment and significantly lowered disease severity; in addition, it promoted alfalfa growth, increasing shoot and root biomass relative to untreated plants. Microscopy and chemical analyses showed that LYZ69 produces cyclic lipopeptides (bacillomycin D and fengycin) that damage hyphal membranes, induce reactive oxygen species accumulation, and trigger apoptosis-like cell death in *Colletotrichum* hyphae. The authors conclude that *B. amyloliquefaciens* LYZ69 is a promising candidate for biological control of anthracnose in alfalfa, providing both disease suppression and plant growth promotion [3].

More broadly, a fungicide-sensitivity study on *C. destructivum* notes that some reports have demonstrated the effectiveness of biofungicides with *Talaromyces flavus* and that plant breeding efforts are directed toward the development of anthracnose-resistant cultivars. However, it emphasizes that fungicide application remains the main strategy for managing anthracnose disease caused by *Colletotrichum* spp., particularly in seed production, underscoring the need to further develop and integrate biological control agents (BCAs) into practical management [2].

At present, field-validated BCAs against alfalfa anthracnose remain limited; based on currently available studies, LYZ69 represents one of the first detailed demonstrations of high biocontrol efficacy under controlled conditions, with further field trials needed to assess its suitability for commercialization and integration into microbial consortia or seed treatments [3].

Although *B. amyloliquefaciens* LYZ69 has demonstrated strong biocontrol efficacy against *Colletotrichum truncatum* on alfalfa, recent research highlights a broader array of microbial strategies for the same purpose [185,186,187,188]. Additional *Bacillus* species, such as *B. licheniformis* CTCRI EB12 [185] and *B. velezensis* LQ-03 [186], have shown antagonistic effects against *Colletotrichum* spp. by disrupting pathogen structures and inhibiting disease progression. Fungal antagonists, particularly *Trichoderma* spp., alone or in combination with arbuscular mycorrhizal fungi, can further reduce anthracnose incidence by enhancing plant defense enzyme activities and improving soil–plant health, demonstrating synergistic interactions among microbial biocontrol agents [187]. Beyond microbial inoculants, emerging reviews emphasize the utility of combined biocontrol agent formulations and, critically, the application of genome editing technologies such as CRISPR/Cas9 to develop plant cultivars with enhanced resistance to fungal pathogens, offering a promising integrated approach that complements biological control for sustainable disease management [188,189].

### 9.3. Physical Management Practices

Direct evidence for physical control methods such as soil steaming, solarization, hot water treatment, mulching, or UV-C irradiation specifically against alfalfa anthracnose is scarce. Most physical interventions relate indirectly to microclimate management and sanitation rather than lethal thermal or irradiation treatments.

From a practical standpoint, the extension guidance emphasizes environmental conditions that favor anthracnose—warm days with cool nights and heavy dew, and disease peaks between July and September—implying that harvest timing, irrigation management, and stand density adjustments that reduce leaf wetness duration are important physical/managerial levers to limit epidemics. Removal of infected residues and thorough cleaning of machinery before entering clean fields are also physical sanitation practices that reduce inoculum spread [8].

### 9.4. Chemical Control

Chemical control plays a significant, though increasingly scrutinized, role in anthracnose management. In the lucerne disease review, chemical methods are reported as the most frequently applied measures for disease management, but their overuse is associated with selection for fungicide-resistant pathogen populations and environmental hazards [7].

Anthracnose control caused by *Colletotrichum* species largely relies on fungicides such as boscalid, chlorothalonil, and penthiopyrad [2].

In vitro fungicide-sensitivity studies with *C. destructivum* from alfalfa in Serbia have provided detailed comparative data for fungicides with different modes of action. Pyraclostrobin (QoI) and tebuconazole (DMI) showed the strongest inhibition of mycelial growth, with mean EC_50_ values of 0.39 and 0.61 μg mL^−1^, respectively. Azoxystrobin (QoI) and flutriafol (DMI) were less active, with higher mean EC_50_ values (2.83 and 2.11 μg mL^−1^). Chlorothalonil (multi-site) and thiophanate-methyl (MBC) showed only moderate inhibition, with mean EC_50_ values of 35.31 and 62.83 μg mL^−1^, and some isolates were resistant or highly resistant to thiophanate-methyl. SDHI fungicides boscalid and fluxapyroxad showed heterogeneous sensitivity: thirteen isolates were sensitive (mean EC_50_ 0.49 and 0.19 μg mL^−1^), but the remaining isolates, including a reference isolate, were highly resistant, with normal growth even at 1000 μg mL^−1^. The authors conclude that, despite their favorable environmental and health profile and efficacy against some other fungi, these SDHIs cannot be considered reliable agents for alfalfa anthracnose control because of the high frequency of resistant isolates [2].

Hu et al. [3] compared the performance of chlorothalonil with *B. amyloliquefaciens* LYZ69 and a second BCA (GB03) under greenhouse conditions. Chlorothalonil provided good control, reducing disease incidence to 22.22% and achieving 95.51% control efficacy; LYZ69 achieved 82.59% control efficacy, while GB03 was considerably less effective.

This positions chlorothalonil as a strong benchmark fungicide in seedling protection [3], although resistance risks and non-target effects associated with intensive chemical use need consideration [2,7].

The fungicide-sensitivity review also emphasizes that resistance to fungicides is a critical challenge in plant pathogen management, with *Colletotrichum* species exhibiting resistance to MBC, DMI, QoI, and SDHI fungicides already reported in various crops [2,190,191,192,193,194]. To reduce the risk of fungicide resistance development in *Colletotrichum* populations, integrated fungicide rotation strategies that alternate modes of action—such as the use of single-site inhibitors (e.g., QoI and DMI fungicides) in combination or alternation with multisite protectants (e.g., chlorothalonil) or fungicides with unrelated mechanisms—have been widely recommended as part of integrated disease management programs [195]. For example, alternating QoI fungicides with multisite contact fungicides and incorporating fungicides from low-risk groups in rotation minimizes consecutive use of high-risk chemistries, while combining seed treatments with foliar sprays optimizes protection throughout the crop cycle. These strategies should be implemented alongside sound cultural practices, including crop rotation, removal of infected residues, optimized cutting regimes, and the deployment of resistant cultivars, within an integrated pest management (IPM) framework. The integration of host resistance, epidemiology-based disease forecasting, and judicious fungicide use into decision-support systems enables precise timing of interventions and minimizes unnecessary chemical inputs, thereby reducing selection pressure for fungicide-resistant *Colletotrichum* populations [99,126]. Practical IPM guidelines for alfalfa further emphasize that combining resistant cultivars with adaptive agronomic practices is the most effective long-term strategy for managing anthracnose and other foliar and stem diseases of alfalfa across diverse production systems [196].

Given the documented risk of fungicide resistance and environmental impacts [2,7], chemical control of alfalfa anthracnose should be embedded in an integrated resistance-management strategy, including rotation of modes of action and avoidance of unnecessary applications (especially where resistant cultivars and BCAs already provide good control), and monitoring efficacy over time.

## 10. Conclusions

Based on the available data, alfalfa is a host to 14 species within the genus *Colletotrichum*. These species exhibit different ecological strategies, allowing them to function as saprobes, endophytes, or pathogens, depending on environmental conditions. This ecological plasticity enables them to transition from a symbiotic lifestyle to pathogenic behavior under conditions that reduce host vigor, including stress or weakened plant health.

For accurate identification and classification of *Colletotrichum* species, a polyphasic approach is recommended, combining morphological characteristics, multigenic phylogenetic analyses, and geographical or ecological data. Further research is necessary to clarify the taxonomic status of these species and establish a robust classification framework.

Although new species of *Colletotrichum* have been identified in recent studies, their introduction requires careful consideration. Many of these species exhibit cryptic morphologies, minimal genetic differences, and often cluster within the same clade with minor genetic deviations. As a result, taxonomists may classify these species as separate entities, even though they could represent the same species. Recently, many species have been synonymized under older taxa.

To mitigate global losses caused by this disease, comprehensive management strategies are essential, including the use of resistant cultivars, crop rotation, and integrated disease control. Alfalfa anthracnose management relies on an integrated approach that includes cultural and ecological practices, host resistance, biological control agents, diagnostic measures, and careful fungicide use. In most production systems, resistant cultivars are the primary control measure, while fungicides and other strategies are employed when resistance is incomplete or when pathogen populations are changing.

Despite substantial progress, major controversies remain regarding *Colletotrichum* species causing alfalfa anthracnose, including conflicting evidence on the pathogenicity of specific taxa (e.g., *C. spinaciae*), unresolved species boundaries within cryptic complexes, inconsistent assignment of primary versus secondary pathogenic roles, and the limited reliability of morphology-based diagnostics across regions. Addressing these gaps will require future research focused on molecular determinants of pathogenic differentiation within species complexes, rapid detection of latent infections, breeding of alfalfa cultivars with broad-spectrum and durable resistance, as well as the integration of population genomics, refined epidemiological modeling for decision-support systems, and studies of microbiome–pathogen–host interactions to improve sustainable disease management.

## Figures and Tables

**Table 1:** Country-level distribution and diversity of *Colletotrichum* species reported to cause anthracnose of alfalfa.

No.	Country	Number of Established Species	Established Species	References
1	USA	9	*C. americae-borealis*	[29]
			*C. dematium*	[61,70]
			*C. destructivum*	[60,61,67]
			*C. gloeosporioides*	[67,70]
			*C. graminicola*	[60,67]
			*C. lini*	[29]
			*C. sojae*	[74]
			*C. trifolii*	[8,14,59,60,61,62,63,64,65,66,67,68,69,71,73]
			*C. truncatum*	[60,65,67]
2	China	5	*C. americae-borealis*	[23,57]
			*C. lini*	[20]
			*C. tofieldiae*	[58]
			*C. trifolii*	[11]
			*C. truncatum*	[11]
3	South Africa	5	*C. coccodes*	[81]
			*C. dematium*	[81]
			*C. destructivum*	[79,80,81]
			*C. trifolii*	[81]
			*C. truncatum*	[81]
4	Argentina	4	*C. americae-borealis*	[21]
			*C. destructivum*	[19]
			*C. trifolii*	[65]
			*C. truncatum*	[65]
5	Netherlands	4	*C. spinaciae*	[49]
			*C. tofieldiae*	[49]
			*C. trifolii*	[84]
			*C. truncatum*	[48]
6	Canada	3	*C. destructivum*	[76,78]
			*C. lindemuthianum*	[75]
			*C. trifolii*	[77,78]
7	Italy	3	*C. dematium*	[27]
			*C. destructivum*	[29]
			*C. trifolii*	[28]
8	Serbia	3	*C. destructivum*	[29,36,46]
			*C. lini*	[44]
			*C. trifolii*	[10,35,36,37,43,46]
9	Australia	2	*C. trifolii*	[13,55,65]
			*C. truncatum*	[65]
10	Croatia	2	*C. destructivum*	[31]
			*C. trifolii*	[30]
11	New Zealand	2	*C. coccodes*	[51]
			*C. lini*	[53,83]
12	Czech Republic	1	*C. trifolii*	[28]
13	France	1	*C. trifolii*	[28]
14	Montenegro	1	*C. trifolii*	[47]
15	Turkey	1	*C. truncatum*	[22]
16	Bulgaria	1	*C. trifolii*	[32]
17	Slovakia	1	*C. trifolii*	[32]
18	Germany	1	*C. trifolii*	[6]
19	United Kingdom	1	*C. dematium*	[34]
20	Russia	1	*C. trifolii*	[28]
21	Switzerland	1	*C. trifolii*	[6]
22	India	1	*C. medicaginis*	[50]
23	Oman	1	*C. lindemuthianum*	[51]
24	Saudi Arabia	1	*C. destructivum*	[24,29,53]
25	Japan	1	*C. trifolii*	[54,55]
26	Iran	1	*C. truncatum*	[56]
27	Israel	1	*C. truncatum*	[49]
28	Morocco	1	*C. destructivum*	[29,82]

**Table 2:** List of confirmed *Colletotrichum* species causing anthracnose of alfalfa, their species complex affiliation, and occurrence.

No.	Species	Complex Species	Occurrence
1	*Colletotrichum americae-borealis*	*C. destructivum*	Localized
2	*Colletotrichum coccodes*	Singleton species	Sporadic
3	*Colletotrichum dematium*	*C. dematium*	Dominant
4	*Colletotrichum destructivum*	*C. destructivum*	Dominant
5	*Colletotrichum gloeosporioides*	*C. gloeosporioides*	Localized
6	*Colletotrichum graminicola*	*C. graminicola*	Sporadic
7	*Colletotrichum lindemuthianum*	*C. orbiculare*	Sporadic
8	*Colletotrichum lini*	*C. destructivum*	Localized
9	*Colletotrichum medicaginis*	*C. orbiculare*	Localized
10	*Colletotrichum sojae*	*C. orchidearum*	Sporadic
11	*Colletotrichum spinaciae*	*C. dematium*	Sporadic
12	*Colletotrichum tofieldiae*	*C. spaethianum*	Localized
13	*Colletotrichum trifolii*	*C. orbiculare*	Dominant
14	*Colletotrichum truncatum*	*C. truncatum*	Dominant

**Table 3:** List of genes, primer pairs, their sequences, amplicon length, and references for the identification of *Colletotrichum* species on alfalfa.

Gene	Primer Name	Sequence	Reference	Identification
*ACT* (Actin)	ACT-512F ACT-783R	5′–ATGTGCAAGGCCGGTTTCGC–3′ 5′–TACGAGTCCTTCTGGCCCAT–3′	[161]	*C. gloeosporioides*; *C. americae-borealis*; *C. lini*; *C. destructivum*; *C. sojae*; *C. trifolii*; *C. dematium*
*CAL* (Calmodulin)	CL1 CL2A CL1C CL2C	5′–GARTWCAAGGAGGCCTTCTC–3′ 5′–TTTTTGCATCATGAGTTGGAC–3′ 5′–GAATTCAAGGAGGCCTTCTC–3′ 5′–CTTCTGCATCATGAGCTGGAC–3′	[162] [108]	*C. gloeosporioides*
*CHS-1* (Chitin synthase)	CHS-79F CHS-345R	5′–TGGGGCAAGGATGCTTGGAAGAAG–3′ 5′–TGGAAGAACCATCTGTGAGAGTTG–3′	[161]	*C. americae-borealis*; *C. lini*; *C. dematium*
*GAPDH* (Glyceraldehyde-3-phosphate dehydrogenase)	GDF GDR	5′–GCCGTCAACGACCCCTTCATTGA–3′ 5′–GGGTGGAGTCGTACTTGAGCATGT–3′	[163]	*C. gloeosporioides*; *C. trifolii*; *C. dematium*
*GS* (Glutamine synthetase)	GSF1 GSR1 GSF3 GSR2	5′–ATGGCCGAGTACATCTGG–3′ 5′–GAACCGTCGAAGTTCCAG–3′ 5′–GCCGGTGGAGGAACCGTCG–3′ 5′–GAACCGTCGAAGTTCCAC–3′	[164] [108]	*C. gloeosporioides*; *C. trifolii*; *C. orbiculare*
*HIS3* (Histone H3)	CYLH-3F CYLH-3R	5′–AGGTCCACTGGTGGCAAG–3′ 5′–AGCTGGATGTCCTTGGACTG–3′	[165]	*C. americae-borealis*; *C. lini*; *C. destructivum*; *C. sojae*; *C. trifolii*
*ITS* (Internal transcribed spacer)	ITS1 ITS4 ITS-1F ITS4	5′–TCCGTAGGTGAACCTGCGG–3′ 5′–TTCTTTTCCTCCGCTTATTGATATGC–3′ 5′–CTTGGTCATTTAGAGGAAGTAA–3′ 5′–TCCTCCGCTTATTGATATGC–3′	[166] [166] [170] [166]	*C. gloeosporioide*; *C. destructivum*; *C. dematium*
*TUB2* (β-Tubulin 2)	T1 Bt-2b T1 BT4R	5′–AACATGCGTGAGATTGTAAGT–3′ 5′–ACCCTCAGTGTAGTGACCCTTGGC–3′ 5′–AACATGCGTGAGATTGTAAGT–3′ 5′–CCRGAYTGRCCRAARACRAAG–3′	[167] [169] [167] [168]	*C. gloeosporioides*; *C. americae-borealis*; *C. lini*; *C. destructivum*; *C. sojae*; *C. graminicola*; *C. coccodes*; *C. trifolii*; *C. dematium*

**Table 4:** Species-core identification genes and PCR primers used for molecular identification of *Colletotrichum* species associated with anthracnose of alfalfa.

No.	Species	Species-Core Identification Genes	Species-Core Identification Primers	References
1	*C. americae-borealis*	*ITS*	ITS1 + ITS4	[23,29,57]
			V9G + ITS-4	[29]
		*GAPDH*	GDF1 + GDR1	[21,29,57]
		*TUB2*	T1 + Bt-2b	[29]
		*CHS-1*	CHS-79F + CHS-354R	[23,29,57]
		*HIS 3*	CYLH3F + CYLH3R	[21,23,29]
		*ACT*	ACT-512F + ACT-783R	[21,23,29]
2	*C. coccodes*	*ITS*	ITS-1 + ITS-4	[29,175]
		*TUB2*	T1 + Bt-2b	[176,177]
		*GAPDH*	GDF1 + GDR1	[176,178]
		*CHS-1*	CHS-79F + CHS-354R	[176]
		*HIS 3*	CYLH3F + CYLH3R	[176]
		*ACT*	ACT-512F + ACT-783R	[176,177,178]
3	*C. dematium*	*ITS*	V9G + ITS-4	[49,73,178]
		*GAPDHA*	GDF1 + GDR1	[49,73,178]
		*CT*	ACT-512F + ACT-783R	[49,73,178]
		*CHS-1*	CHS-79F + CHS-354R	[49,73]
		*TUB2*	BT2Fd + BT4R	[49,73,178]
			T1 + Bt-2b	[49,178]
		*HIS3*	CYLH3F + CYLH3R	[49,73]
4	*C. destructivum*	*ITS*	ITS1 + ITS4	[29,73]
			ITS1F + ITS4	[29]
		*GAPDH*	GDF1 + GDR1	[29,73]
		*TUB2*	T1 + Bt-2b	[29,73]
			T1 + BT4R	[29]
		*CHS-1*	CHS-79F + CHS-354R	[29,73]
		*HIS 3*	CYLH3F + CYLH3R	[29,73]
		*ACT*	ACT-512F + ACT-783R	[29,73]
5	*C. gloeosporioides*	*ITS*	ITS-1F + ITS-4	[74,118,119]
			ITS-5 + ITS-4	[175]
		*GAPDH*	GDF1 + GDR1	[74,119,178]
		*CHS-1*	CHS-79F + CHS-354R	[74,119]
		*HIS3*	CYLH3F + CYLH3R	[74,119]
		*ACT*	ACT-512F + ACT-783R	[74,119,178]
		*TUB2*	BT2Fd+ BT4R	[74,119,178]
			T1 + Bt-2b	[74,119,178]
		*GS*	GSF1 + GSR1	[119]
6	*C. graminicola*	*ITS*	ITS-1 + ITS-4	[73,175]
		*CHS-1*	CHS-79F + CHS-354R	[73]
		*ACT*	ACT-512F + ACT-783R	[73]
		*TUB2*	BT2Fd+ BT4R	[73]
			T1 + Bt-2b	[73]
7	*C. lindemuthianum*	*ITS*	ITS-1F + ITS-4	[73,119]
		*GAPDH*	GDF1 + GDR1	[73,119]
		*CHS-1*	CHS-79F + CHS-354R	[73,119]
		*HIS3*	CYLH3F + CYLH3R	[73,119]
		*ACT*	ACT-512F + ACT-783R	[73,119]
		*TUB2*	BT2Fd+ BT4R	[73,119]
			T1 + Bt-2b	[73,119]
		*GS*	GSF1 + GSR1	[73,119]
8	*C. lini*	*ITS*	ITS1 + ITS4	[29,73]
			ITS1F + ITS4	[29,73]
		*GAPDH*	GDF1 + GDR1	[29,73]
		*TUB2*	T1 + Bt-2b	[29,73]
			T1 + BT4R	[29,73]
		*CHS-1*	CHS-79F + CHS-354R	[29,73]
		*HIS 3*	CYLH3F + CYLH3R	[29,73]
		*ACT*	ACT-512F + ACT-783R	[29,73]
9	*C. medicaginis*	*ITS*	ITS1 + ITS4	[57,74]
			ITS-1F + ITS4	[57,74]
		*GAPDH*	GDF1 + GDR1	[57,74]
10	*C. sojae*	*ITS*	ITS1 + ITS4	[57,74]
			ITS-1F + ITS4	[57,74]
		*GAPDH*	GDF1 + GDR1	[57,74]
		*TUB2*	T1 + Bt-2b	[57,74]
			T1 + BT4R	[74]
		*HIS 3*	CYLH3F + CYLH3R	[57,74]
		*ACT*	ACT-512F + ACT-783R	[57,74]
		*CHS-1*	CHS-79F + CHS-354R	[74]
11	*C. spinaciae*	*ITS*	V9G + ITS-4	[49,73]
		*GAPDH*	GDF1 + GDR1	[49,73]
		*ACT*	ACT-512F + ACT-783R	[49,73]
		*CHS-1*	CHS-79F + CHS-354R	[49,73]
		*TUB2*	BT2Fd + BT4R	[49,73]
			T1 + Bt-2b	[49,73]
		*HIS3*	CYLH3F + CYLH3R	[49,73]
12	*C. tofieldiae*	*ITS*	V9G + ITS-4	[49,73]
		*GAPDH*	GDF1 + GDR1	[49,73]
		*ACT*	ACT-512F + ACT-783R	[49,73]
		*CHS-1*	CHS-79F + CHS-354R	[49,73]
		*TUB2*	BT2Fd + BT4R	[49,73]
			T1 + Bt-2b	[49,73]
		*HIS3*	CYLH3F + CYLH3R	[49]
13	*C. trifolii*	*ITS*	ITS-1F + ITS-4	[73,119]
		*GAPDH*	GDF1 + GDR1	[73,119]
		*CHS-1*	CHS-79F + CHS-354R	[73,119]
		*HIS3*	CYLH3F + CYLH3R	[73,119]
		*ACT*	ACT-512F + ACT-783R	[73,119]
		*TUB2*	BT2Fd+ BT4R	[73,119]
			T1 + Bt-2b	[73,119]
		*GS*	GSF1 + GSR1	[73,119]
14	*C. truncatum*	*ITS*	V9G + ITS-4	[49,73,178]
		*GAPDH*	GDF1 + GDR1	[49,73,178]
		*ACT*	ACT-512F + ACT-783R	[49,73,178]
		*CHS-1*	CHS-79F + CHS-354R	[49,73]
		*TUB2*	BT2Fd + BT4R	[49,73,178]
			T1 + Bt-2b	[49,73,178]
		*HIS3*	CYLH3F + CYLH3R	[49]

## Data Availability

No new data were created or analyzed in this study. Data sharing is not applicable to this article.
